# MMP-13 stimulates osteoclast differentiation and activation in tumour breast bone metastases

**DOI:** 10.1186/bcr3047

**Published:** 2011-10-27

**Authors:** Eliana Pivetta, Martina Scapolan, Marina Pecolo, Bruna Wassermann, Imad Abu-Rumeileh, Luca Balestreri, Eugenio Borsatti, Claudio Tripodo, Alfonso Colombatti, Paola Spessotto

**Affiliations:** 1 Experimental Oncology 2, CRO, IRCCS, National Cancer Institute, Via Franco Gallini 2, 33081 Aviano (PN), Italy; 2 Division of Radiology, CRO, IRCCS, National Cancer Institute, Via Franco Gallini 2, 33081 Aviano (PN), Italy; 3 Division of Nuclear Medicine, CRO, IRCCS, National Cancer Institute, Via Franco Gallini 2, 33081 Aviano (PN), Italy; 4 Department of Health Science, Human Pathology Section, University of Palermo, School of Medicine Via del Vespro 129, 90127 Palermo, Italy; 5 Department of Biomedical Science and Technology, University of Udine, Piazzale Massimiliano Kolbe 4, 33100 Udine, Italy; 6 MATI (Microgravity, Ageing, Training, Immobility) Excellence Centre, University of Udine, Piazzale Massimiliano Kolbe 4, 33100 Udine, Italy

## Abstract

**Introduction:**

The increased bone degradation in osteolytic metastases depends on stimulation of mature osteoclasts and on continuous differentiation of new pre-osteoclasts. Metalloproteinases (MMP)-13 is expressed in a broad range of primary malignant tumours and it is emerging as a novel biomarker. Recent data suggest a direct role of MMP-13 in dissolving bone matrix complementing the activity of MMP-9 and other enzymes. Tumour-microenvironment interactions alter gene expression in malignant breast tumour cells promoting osteolytic bone metastasis. Gene expression profiles revealed that *MMP-13 *was among the up-regulated genes in tumour-bone interface and its abrogation reduced bone erosion. The precise mechanism remained not fully understood. Our purpose was to further investigate the mechanistic role of MMP-13 in bone osteolytic lesions.

**Methods:**

MDA-MB-231 breast cancer cells that express MMP-13 were used as a model for *in vitro *and *in vivo *experiments. Conditioned media from MDA-MB-231 cells were added to peripheral blood mononuclear cultures to monitor pre-osteoclast differentiation and activation. Bone erosion was evaluated after injection of MMP-13-silenced MDA-MB-231 cells into nude mice femurs.

**Results:**

MMP-13 was co-expressed by human breast tumour bone metastases with its activator MT1-MMP. MMP-13 was up-regulated in breast cancer cells after *in vitro *stimulation with IL-8 and was responsible for increased bone resorption and osteoclastogenesis, both of which were reduced by MMP inhibitors. We hypothesized that MMP-13 might be directly involved in the loop promoting pre-osteoclast differentiation and activity. We obtained further evidence for a direct role of MMP-13 in bone metastasis by a silencing approach: conditioned media from MDA-MB-231 after MMP-13 abrogation or co-cultivation of silenced cells with pre-osteoclast were unable to increase pre-osteoclast differentiation and resorption activity. MMP-13 activated pre-MMP-9 and promoted the cleavage of galectin-3, a suppressor of osteoclastogenesis, thus contributing to pre-osteoclast differentiation. Accordingly, MMP-13 abrogation in tumour cells injected into the femurs of nude mice reduced the differentiation of TRAP positive cells in bone marrow and within the tumour mass as well as bone erosion.

**Conclusions:**

These results indicate that within the inflammatory bone microenvironment MMP-13 production was up-regulated in breast tumour cells leading to increased pre-osteoclast differentiation and their subsequent activation.

## Introduction

Bone metastases are the most frequent complication in breast cancer and lead to severe disease and pain [1]. The development of osteolytic metastases depends on the tropism of breast cancer cells for bone that is the result of their ability to migrate, intravasate, extravasate, and finally to thrive in the metastatic site where osteoclasts (OCs) form lytic lesions through the activation of a complex cascade of morphological and biochemical changes and release of growth factors sequestered in the bone matrix. Breast cancer cells that metastasize to bone establish a tight interaction with the marrow microenvironment and express several classes of molecules that modulate tumour-bone interplays. Among these are chemokines and chemokine receptors, growth factors, cell adhesion molecules involved in invasion and metalloproteinases (MMPs) that play a pivotal role in bone degradation. Recent data suggest a direct role of MMP-13 in dissolving bone matrix, an osteolytic activity complementing MMP-9 and other enzymes [2]. MMP-13 was originally identified from a cDNA library derived from a breast carcinoma [3] and subsequently found to be produced by tumours of different sources [4]. It is synthesized as a proenzyme and then activated by MT1-MMP; indeed both these enzymes co-localize in several human malignant tumours [5]. The levels of MMP-13 expression depend on the exposure to a variety of factors, including hormones and cytokines, present in the bone microenvironment, such as PTH and PTHrP [6,7]. MMP-13 is up-regulated by IL-1 α, -β, and transforming growth factor (TGF)-β in several human malignancies [4] and higher expression of MMP-13 is associated with increased malignancy [8-10] and shorter overall survival [11,12]. However, while MMP-13 might represent a poor prognosis marker in breast carcinomas [13] it seems unlikely that tumour aggressiveness and bone metastatic lesions solely depend on its digestive function in the bone microenvironment. Singh and collaborators applied micro-dissection to breast tumour-bone interface and found that *MMP-13*, *receptor activator of nuclear factor kappa-B ligand (RANKL) *and *integrin binding sialoprotein *were among the most up-regulated genes [14]. They further demonstrated that down-regulation of *MMP-13 *with antisense oligonucleotides significantly reduced bone destruction.

We thus hypothesized that MMP-13 might be involved in the complex network of interactions between tumour and bone cells promoting not only OC bone-destructive activity, but also OC differentiation. Here, we demonstrated the functional involvement of MMP-13 in breast cancer bone metastasis: MMP-13 activated pre-MMP-9 and cleaved galectin-3 on OC precursors. These actions resulted in stimulation of mature OC digestive ability as well as in enhanced differentiation of OC precursors.

## Materials and methods

### Reagents

Recombinant human IL-8, Parathyroid Hormone-related Protein (PTHrP), Macrophage Colony-Stimulating Factor (M-CSF) and soluble RANKL were purchased from Peprotech (London, UK). Recombinant human MMP-13 (catalogue #BML-SE493-0010, lot #3-H4512a) was obtained from Enzo Life Sciences Inc. (Farmingdale, NY, USA). MMP-13 specific inhibitor CL-82198 was purchased from Calbiochem (La Jolla, CA, USA), metalloproteases generic inhibitor GM6001 was obtained from Chemicon (Chemicon International, Temecula, CA, USA). Rabbit polyclonal anti-cow Cytokeratin Wide Spectrum Screening (catalogue #Z0622) was from Dako (DakoCytomation Inc, Carpinteria, CA, USA). Mouse anti-human MMP-13 (catalogue #MAB13442), MMP-9 (catalogue #MAB13458) and MMP-2 (catalogue #MAB13406) were purchased from Chemicon (Chemicon International); mouse anti-human MT1-MMP (catalogue #MAB-10765) and mouse anti-human TIMP-1 (catalogue #MAB-12070) were purchased from Immunological Science (Rome, Italy). Mouse anti-human tubulin (catalogue #T 6074) was obtained from Sigma (Milan, Italy).

### Cells

Human breast adenocarcinoma cell line MDA-MB-231 was maintained in DMEM (BioWhittaker, Lonza, Milan, Italy) with 10% fetal calf serum (FCS). Human breast adenocarcinoma cell line MCF7 (ATCC, Rockville, MD, USA) was maintained in DMEM with 10% FCS, insulin (10 μg/ml), sodium pyruvate (1 mM) and non-essential amino acids (0.1 mM).

### Human OC cultures

Peripheral blood mononuclear cells (PBMCs) were isolated from buffy coat preparations obtained from the Blood Bank of the CRO-IRCCS, National Cancer Institute, Aviano, Italy as previously described [15]. All procedures were performed with written informed consent according to the Declaration of Helsinki and used a protocol approved by the Scientific Director of the Institute. Cells were grown in Roswell Park Memorial Institute medium, with 10% FCS, and osteoclastogenesis was induced for the first three days of culture with human M-CSF (30 ng/ml) and human RANKL (40 ng/ml). At Day 4 pre-OCs were cultured with complete medium containing M-CSF plus RANKL or only with concentrated MDA-MB-231 conditioned media.

### Conditioned medium (CM) preparation

MDA-MB-231 cells, grown until sub-confluency, were starved or stimulated with IL-8 (10 ng/ml) or PTHrP (100 ng/ml) in serum free DMEM, for 24 h. CM were then collected, centrifuged and concentrated, aliquoted and stored at -20°C until use.

### TRAP staining

To quantify the formation of Tartrate-Resistant Acid Phosphatase positive (TRAP) multinucleated cells, PBMC cultures and paraffin-embedded sections were stained for TRAP using a Leukocyte Acid Phosphatase kit (Sigma Diagnostics, Milan, Italy), according to the manufacturer's instructions. Cells positive for TRAP and having more than three nuclei were considered as TRAP positive multinucleated OCs.

### Bone resorption assay

PBMCs were seeded onto calcium phosphate coated wells (Biocoat, 16-well Osteologic Multitest Slide, BD Biosciences, Buccinasco, Italy) and cultured for up to seven days in different culture conditions. Cells were removed by bleach treatment in order to observe resorption pits under light microscope.

### Computer-assisted morphometric analyses

To quantitatively evaluate OC resorption activity, computer-assisted morphometric analyses were performed on the images acquired with Nikon Eclipse TS100 microscope (Melville, NY, USA) equipped with a Canon camera (Canon USA, Inc., Jamesburg, NJ, USA) by using the ImageJ software (National Institute of Health, Bethesda, MD, USA) [16]. Images of TRAP or immune-stained bone sections were captured with a Leica ICC50 camera connected with a Leica DM 750 microscope (Leica Microsystems Heidelberg, Mannheim, Germany) equipped with N Plan objective 5×/-0.12 NA, HI-Plan objective 10×/0.25 NA and objective 20×, all from Leica. The images were then evaluated by ImageJ computer-assisted morphometric analysis (National Institute of Health, Bethesda, MD, USA).

### Immunohistochemistry

**Figure 1:**
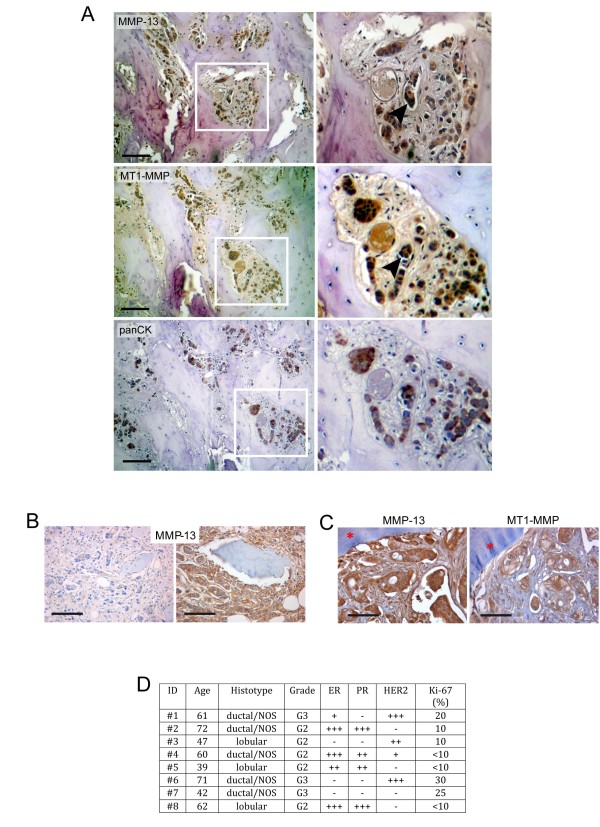
**MMP-13 and MT1-MMP are expressed in human breast cancer bone metastasis**. **A**. Representative immunohistochemical images of MMP-13 and MT1-MMP expression in serial paraffin-embedded human bone metastasis. The positive staining of the two enzymes is associated to tumour cells as indicated by co-localization with pan-citokeratins (lower panel). Magnification of the insets is provided on the right. The black arrowheads in the insets of MMP-13 and MT1-MMP immunostaining indicate a cluster of cancer cells in the bone marrow inter-trabecular space, which express both MMP-13 and MT1-MMP. Scale bar = 100 μm. **B**. MMP-13 expression in bone metastases from invasive ductal breast cancer. MMP-13 expression is detected on neoplastic cells with a variable staining intensity ranging from slight (left) to marked (right). Scale bar = 100 μm. **C**. MMP-13 and MT1-MMP are associated in metastatic invasive breast cancer cells at tumour-bone interface (red asterisk). Scale bar = 50 μm. **D**. Histotype and grade status, ER, PR, HER2 and Ki-67 positivity for each patient whose bone metastases were examined for MMP-13 and MT1-MMP expression by immunohistochemistry.

Human bone metastasis paraffin sections have been gathered from the Archieves of the Human Pathology Section, School of Medicine, University of Palermo (Italy). All the samples were processed and handled according to the Institutions' Ethical Guidelines. In Figure 1C, the histotype and grade status, ER, PR, HER2 and Ki-67 positivity for each patient are reported.

After deparaffination and rehydration using standard methodologies, primary antibodies were applied. HRP-conjugated secondary antibodies (Amersham, GE Healthcare Europe GmbH, Milan, Italy) were used at a 1:100 dilution for one hour at room temperature. Visualization was achieved with diaminobenzidine (DAB) substrate (Vector Laboratories, Burlingame, CA, USA). Samples were counterstained with hematoxylin, dehydrated and mounted.

### Western immunoblotting

Supernatants and cell lysates obtained from cell culture samples were resolved in a SDS-polyacrylamide gel (4 to 20% polyacrylamide gradient) (Bio-Rad; Milan, Italy) under reducing conditions and transferred to a nitrocellulose membrane. The membranes were saturated with tris-buffered saline (TBS) buffer (20 mM TRIS and 0.15 M NaCl) containing 0.1% Tween-20 and 5% not fat dry milk for one hour at room temperature and then incubated with primary antibodies at 4°C overnight. The membranes were incubated with HRP-conjugated appropriate secondary antibodies (Amersham, GE Healthcare Europe GmbH, Milan, Italy) and then revealed with the ECL Plus chemiluminescence kit (Amersham, GE Healthcare).

### MMP-13 silencing

Human MMP-13 expression was abrogated by stably transfecting MDA-MB-231 with HuSH 29-mer shRNA constructs against MMP-13 (Origene Technologies, Rockville, MD, USA) using Amaxa Cell Line Nucleofector Kit V (Lonza, Koln, Germany) according to the manufacturer's instructions. Negative controls included scrambled non-effective shRNA. The stable clones were selected and maintained in complete medium supplemented with puromycin.

### *In vivo *studies

Procedures involving animals and their care were conducted according to the institutional guidelines in compliance with national laws (D.Lgs. n° 116/92). The Ethic Committee for Animal Experimentation (CESA) of CRO-IRCCS, Aviano (Italy) approved the proposed animal research by protocols #2010/03/05/P1 and #2011/08/03/P1a. Six-week-old female *Foxn1**^nu ^***nude mice (Harlan Laboratories, Udine, Italy) were anaesthetized and the right leg was flexed at 90 degrees. Using a 30 gauge needle a small hole was made into the femoral bone marrow below the patella and was followed by an injection of 2 × 10^5 ^MDA-MB-231 cells suspended in 5 μl of sterile PBS with a Hamilton syringe. Mice were divided into subgroups and inoculated as follows with: PBS (negative control, *n *= 6); MDA-MB-231 wild type cells (positive control, *n *= 8); MDA-MB-231 cells transfected with shRNA vector control (scrambled, *n *= 11); and MDA-MB-231 cells transfected with shRNA against MMP-13 (*n *= 12). A total of 28 days after treatment mice were anaesthetized and analyzed by ultrasound (Siemens-Acuson Sequoia 512, small parts 15 MHz linear transducer, Siemens AG Medical Solutions, Herlangen, Germany) and computed tomography (CT, GE Lightspeed 32-slice CT Scanner; GE Healthcare Europe GmbH, Milan, Italy) in order to observe and quantify tumour masses and developed osteolytic lesions, respectively. The average volume of tumour masses was calculated as follows: V = 0.5 × d_L _× d_S_^2^; d_L_, larger distance; d_S_, smaller distance. All mice were sacrificed and both left and right femurs were collected for immunohistochemical analysis. Tumours were collected for Western blotting analysis.

### Statistical analysis

Statistical significance of the results was determined by using the unpaired and paired Student's *t *test. A value of *P *< 0.05 was considered significant.

## Results

### MMP-13 is expressed by breast cancer cells in human bone osteolytic lesions

Initially we provided the first evidence that MMP-13 protein was expressed in all human metastatic breast bone lytic lesions and its immunohistochemical reactivity ranged from weak to intense (*n *= 8) (Figure 1), irrespective of histotype and grade status, ER, PR, HER2 and Ki-67 positivity of the patients (Figure 1C). MT1-MMP, the MMP-13 natural activator [17], was co-expressed and co-localized with MMP-13 in bone metastatic lesions as confirmed by serial pan-cytokeratins staining (Figure 1).

### MMP-13 secretion is modulated by cytokines and extracellular matrix (ECM) substrates

**Figure 2:**
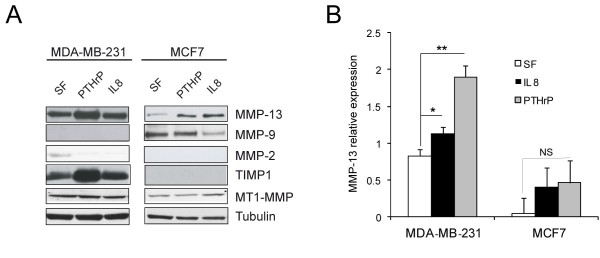
**MMP expression in MDA-MB-231 and MCF7 cells**. **A**. MMP expression in MDA-MB-231 and MCF7 cells, stimulated with PTHrP or IL-8 for 24 h in serum free medium. Protein extracts of cellular lysates (for detection of MT1-MMP) and supernatants (for detection of MMP-13, MMP-9, MMP-2 and TIMP-1) were loaded for Western blotting analysis. Representative blottings are reported. Tubulin was used as a loading control. **B**. Quantification of Western blot analysis for MMP-13 expression by QuantityOne software (Bio-Rad; Milan, Italy). Mean values (± SD) of MMP-13 relative expression levels of three different experiments are reported. *P < 0.05; **P < 0.01; NS, not significant.

MDA-MB-231 breast cancer cells secreted higher levels of MMP-13 than less aggressive MCF7 cells (Figure 2). Consistent with the MMP-13 levels, also TIMP-1 expression was up-regulated in MDA-MB-231 cells. MCF7 cells were negative for TIMP-1. We did not detect MMP-1 (data not shown) nor MMP-2 significant levels in MDA-MD-231 and MCF-7 supernatants, whereas MMP-9 expression was observed only in MCF7 cells. Either PTHrP or IL-8 stimulation led to increased secretion of MMP-13 in both cell lines but in a significant manner only in MDA-MB-231 cells (Figure 2).

A panel of ECM molecules was used to evaluate adhesion property of the two breast cancer cell lines that differ in metastatic potential. The highly metastatic MDA-MB-231 cells displayed strong adhesive properties to fibronectin and LN-1 and to substrates typical of the bone microenvironment (collagens I and III), or of basement membranes (collagen type IV) (Additional file 1, Figure S1A, B; Additional file 4, Supplementary materials and methods). The percentage of adherent cells did not significantly change when a higher detaching force (spin off) was applied. Similar, but slightly lower, adhesion forces were detected in the non-invasive MCF7 cell line (Additional file 1, Figure S1A, B; Additional file 4, Supplementary materials and methods). On the contrary, while MDA-MB-231 cells preferentially migrated on fibronectin and collagens, MCF7 migrated very poorly or not at all on all the substrates tested (Additional file 1, Figure S1C, D). Since bone metastatic lesions are accompanied by inflammatory infiltrates in which IL-8 stimulates OC-mediated bone erosions [18] we found that IL-8 stimulation increased significantly the migration of MDA-MB-231 cells on collagens compared to the other ECM molecules: we detected only about 30% increased migration on fibronectin versus 55% increase on collagen I. Moreover, MMP-13 expression was up-regulated in MDA-MB-231 cells by adhesion to collagen III and IL-8 treatment potentiated this effect particularly in cells adherent to collagens I and IV (Additional file 1, Figure S1E; Additional file 4, Supplementary materials and methods). Based on the above findings we used MDA-MD-231 for all the following experiments.

### MMP-13 is involved in osteoclastogenesis as well as in OC activity

**Figure 3:**
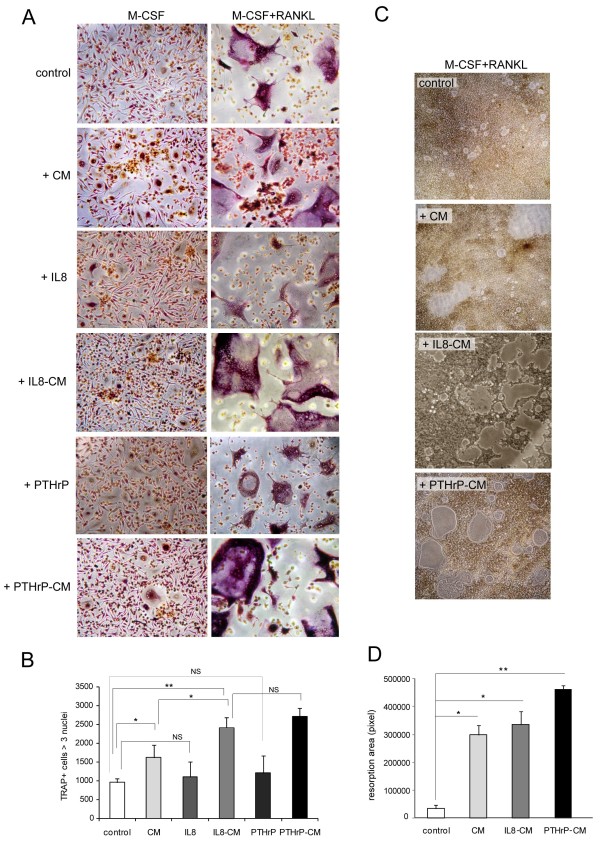
**Breast cancer CM increase osteoclastogenesis and bone resorption**. **A**. Human OC precursors were primed with M-CSF and RANKL or with M-CSF alone for three days and then cultured for another four days with the addition of CM from MDA-MB-231 cells (CM), IL-8- or PTHrP-stimulated cells (IL-8-CM, PTHrP-CM) or with IL-8 (10 ng/ml) and PTHrP (100 ng/ml) alone. Representative images of TRAP staining are shown and the total number of multinucleated TRAP positive cells per well of a 24-well plate is provided (**B**). Mean values ± SD of three experiments are reported. **C**. PBMCs were seeded on Biocoat matrices to evaluate resorption activity. Representative images of resorption pits are shown and the quantification of the total resorption area per well of a 96-well plate is reported as mean values ± SD of three experiments (**D**). *P < 0.05; **P < 0.02; NS, not significant.

Since MDA-MB-231 cells secreted large amounts of MMP-13 and can metastasize to bone where they induce osteolytic lesions, we investigated the role of MMP-13 in OC differentiation treating M-CSF or M-CSF plus RANKL primed PBMCs with CM from MDA-MB-231. When M-CSF alone was used for the first three days, the addition of CM determined only a slight increase in TRAP-positive multinucleated cells; in the presence of M-CSF plus RANKL the number of multinucleated TRAP positive cells was higher than in control cultures. The effect was more pronounced with the addition of CM obtained from MDA-MB-231 cells stimulated with IL-8 and PTHrP (IL-8-CM and PTHrP-CM) (Figure 3A, B). The size of multinucleated OCs also was abundantly increased in cells cultured with CM, especially with IL-8 and PTHrP-CM (Figure 3A). Consequently, resorption was more pronounced in cultures treated with CM or IL-8 and PTHrP-CM (Figure 3C, D). We hypothesized that the extent of resorption could directly depend on the amount of MMP-13 released in the supernatant, since the augmented MMP-13 levels obtained in PTHrP-CM but also in IL-8 treated cells (see Figure 2) increased not only the number of pits but also resulted in larger digestion areas. The addition of IL-8 or PTHrP alone to PBMC cultures in the presence of M-CSF plus RANKL did not significantly increase the number of multinucleated TRAP positive cells (Figure 3A, B).

To determine if MMP-13 expressed by tumour cells and the increased OC differentiation and activation were causally linked, we used two approaches to abolish MMP-13 expression.

**Figure 4:**
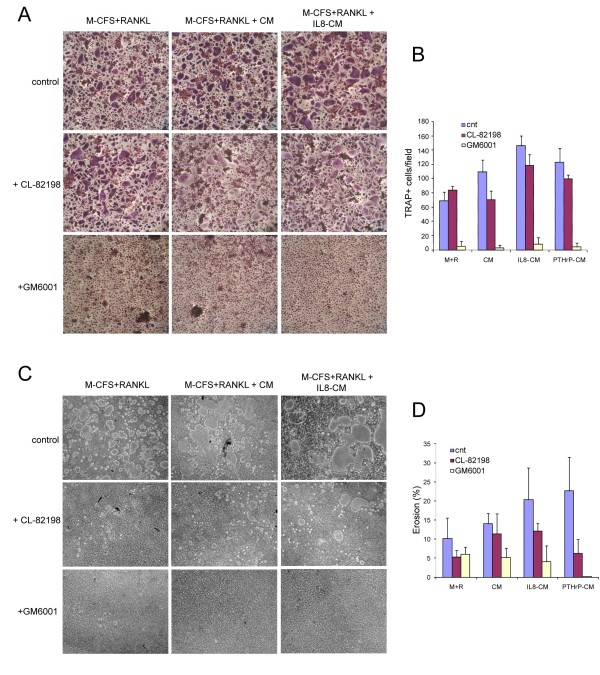
**OC differentiation and resorption are potentiated by the presence of MMPs**. PBMCs were cultured on plastic (**A**) or on Biocoat matrices (**C**) with M-CSF and RANKL for three days and then with the addition of CM, PTHrP-CM or IL-8-CM for further four days. MMP inhibitors (10 μM GM6001 and 10 μg/ml CL-82198) were added at Day 1 of culture. TRAP staining was performed at Day 7. Representative images are shown in (A) and the number of multinucleated TRAP positive cells per field (10×) is reported in (**B**) as the mean ± SD. 8 fields for each experiment (*n *= 3) were examined. C. Representative fields of resorption activity. **D**. Quantification of the resorbed area is reported as the mean values ± SD of the percentage eroded area per well of a 96-well plate. Three experiments were evaluated.

First, in the presence of the MMP-13 specific inhibitor CL-82198 [19,20] or of the generic MMP activity inhibitor GM6001 a lower number of multinucleated TRAP positive cells was detected (Figure 4A, B). The inhibitory effect of GM6001 was very strong and almost totally blocked OC differentiation. Consequently, MMP inhibitors reduced *in vitro *bone resorption, suggesting that this OC activity was due to a large extent to MMPs; in fact, CL-82198 partially and GM6001 fully inhibited resorption (Figure 4C, D). While the specific MMP-13 inhibitor was much less effective, it still reduced the size of resorption pits in OC cultures treated with PTHrP- and IL-8-CM (Figure 4C, D and data not shown).

**Figure 5:**
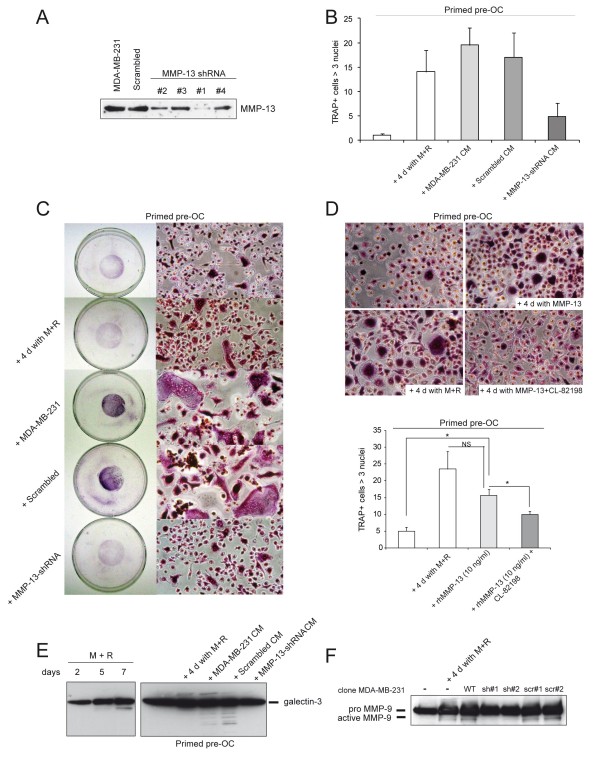
**MMP-13 potentiates OC differentiation**. **A**. Western blotting analysis of MMP-13 expression in supernatants of wild type MDA-MB-231, scrambled shRNA and MMP-13 shRNA (clones #1, #2, #3 and #4) cells. **B**. PBMCs were primed with RANKL and M-CSF (primed pre-OC) for three days and then cultured with the addition of CM, obtained from wild type (MDA-MB-231 CM), scrambled shRNA (scrambled CM) and MMP-13 shRNA cells (clone #1) (MMP-13-shRNA CM) for further four days. The graph reports the mean ± SD of the number of multinucleated TRAP positive cells per field (20×). 10 fields for each experiment (*n *= 3) were examined. **C**. PBMCs were primed with M-CSF and RANKL for three days and then co-cultured with wild type, Scrambled and MMP-13 silenced MDA-MB-231 cells (clone #1) for further four days. Representative images of TRAP staining and their magnifications are reported. **D**. Recombinant human MMP-13 (10 ng/ml) was added alone or together with its specific inhibitor CL-82198 (10 μg/ml) to PBMCs primed with M-CSF and RANKL for three days. Representative images of TRAP staining of OC cultures at Day 7 are shown. The graph reports the mean ± SE of the number of multinucleated TRAP positive cells per field (20×). Five fields for each experiment (*n *= 3) were examined. *P < 0.05; NS, not significant. **E**. Western blotting analysis of galectin-3 in extracts of PBMCs cultured in the presence of RANKL and M-CSF for two, five or seven days (left panel) and of pre-OC cultured with CM as reported in (B) (right panel). **F**. Western blotting analysis for pro- and active MMP-9 forms in primed pre-OC treated as reported in (C) with silenced (sh#1 and sh#2) or Scrambled (scr#1 and scr#2) clones.

In the second approach, MMP-13 expression was suppressed by specific shRNA sequences. OC precursors were treated with CM derived from control or MDA-MB-231 cells transfected with scrambled shMMP-13 or with CM from one of the clones displaying an almost complete protein silencing (clone #1) (Figure 5A). Only the treatment with CM derived from specific MMP-13 shRNA significantly reduced OC number (Figure 5B). Then, M-CSF and RANKL primed PBMCs were co-cultured with MDA-MB-231 cells. TRAP staining of seven-day-old co-cultures showed that abrogation of MMP-13 expression nearly fully abolished the enhanced osteoclastogenesis (Figure 5C). Silenced clones were also stimulated with IL-8 or PTHrP and the corresponding CM added to OC precursors. A slight even, if not significant, increase in osteoclastogenesis was detected (Additional file 2, Figure S1A; Additional file 4, Supplementary materials and methods), indicating that stimulation of IL-8 or PTHrP can induce some MMP-13 secretion in not fully silenced cells. In conclusion, MMP-13 shRNA CM was unable to increase the number of TRAP-positive multinucleated cells compared to control and "scrambled" CM suggesting that MMP-13 effectively potentiated OC differentiation.

From the previous series of experiments we concluded that MMP-13 was involved in both differentiation and activation of OCs. Furthermore, the addition of human recombinant MMP-13 to the PBMC cultures was able to enhance the number of TRAP multinucleated cells, reinforcing the evidence that this metalloproteinase could potentiate OC differentiation process (Figure 5D).

Next, the mechanism of this involvement was investigated on the basis of the following evidence: 1) galectin-3, a known inhibitor of osteoclastogenesis [21], is a substrate of MMP-13 *in vitro *[22]; 2) MMP-13 regulates the activation of pre-MMP-9 [14] that is associated with OC differentiation process [23]. Western blotting of protein extracts revealed that galectin-3 was degraded during ostoclastogenesis (Figure 5E, left panel). When pre-OCs were treated with CM obtained from both control and scrambled MMP-13 MDA-MB-231 cells, galectin-3 resulted fragmented in protein extracts; on the contrary, degradation was absent when MMP-13 shRNA CM were used (Figure 5E, right panel). Finally, we analyzed the presence of MMP-9 in the supernatants of the co-cultures: the active form of MMP-9 was detected to a much greater extent in differentiated OC cultures and it was almost totally absent when silenced cells were added to pre-OCs (Figure 5F).

### MMP-13 increases osteoclastogenic potential *in vivo*

Next, the *in vitro *MMP-13 silenced breast tumour model results were checked *in vivo*. First, we excluded the transfection with shRNAs influenced cell growth. All cells examined displayed growth curves with a very similar trend (Additional file 2, Figure S1B; Additional file 4, Supplementary materials and methods). Conversely, only MMP-13 shRNA clones migrated significantly less towards collagen type I respect to WT and scrambled MMP-13 cells, indicating that MMP-13 silencing was effective [24-26] (Additional file 2, Figure S1C; Additional file 4, Supplementary materials and methods).

**Figure 6:**
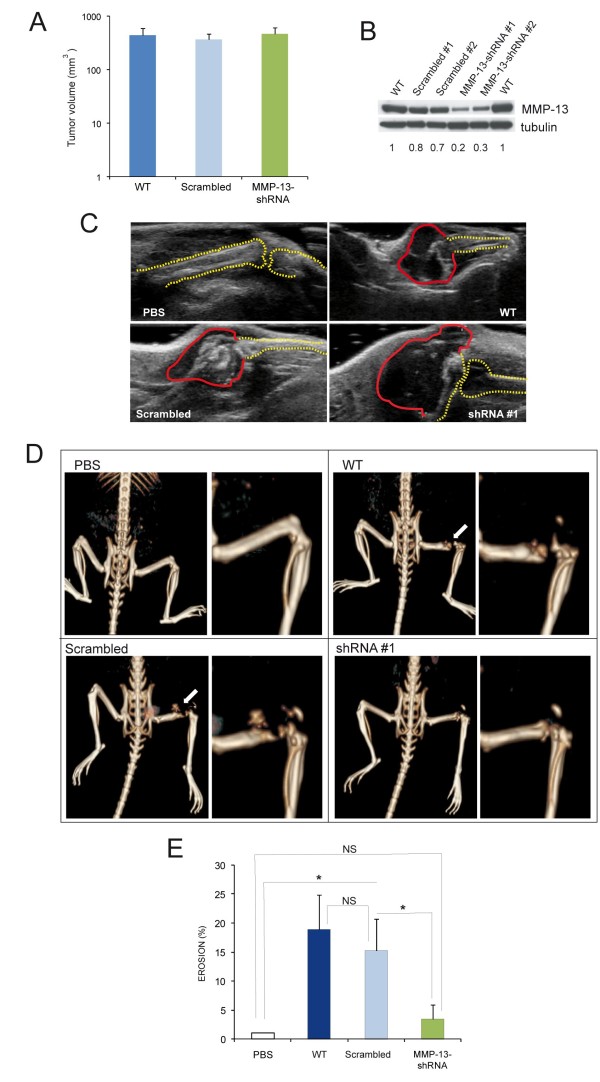
**MMP-13 increases osteoclastogenic potential *in vivo***. WT and transfected cells were inoculated into the femurs of 6 weeks-old nude mice. Volume (**A**) and MMP-13 expression (**B**) of the tumours developed one month after the inoculation of WT, scrambled and shRNA MDA-MB-231 cell clones. Data in (A) are expressed as the mean ± SE (WT, *n *= 8; Scrambled, *n *= 11; MMP-13-shRNA, *n *= 12). **C**. Representative ultrasound images of WT, scrambled and shRNA #1 clones after one month from injection into nude mice. The basal skeletal condition is represented by PBS inoculation. Red and yellow dotted lines approximately label tumour mass and bone profile, respectively. **D**. Representative images and their magnification of CT analysis of skeletal lesions produced by MDA-MB-231 cell clones injected into nude mice. **E**. The graph shows the level of femur erosion, calculated as follows: (1- (length of the undamaged femur injected/length of untreated counterpart)) × 100. Results are the mean ± SE (WT, *n *= 8; Scrambled, *n *= 11; MMP-13-shRNA, *n *= 12). *P < 0.05; NS, not significant.

**Figure 7:**
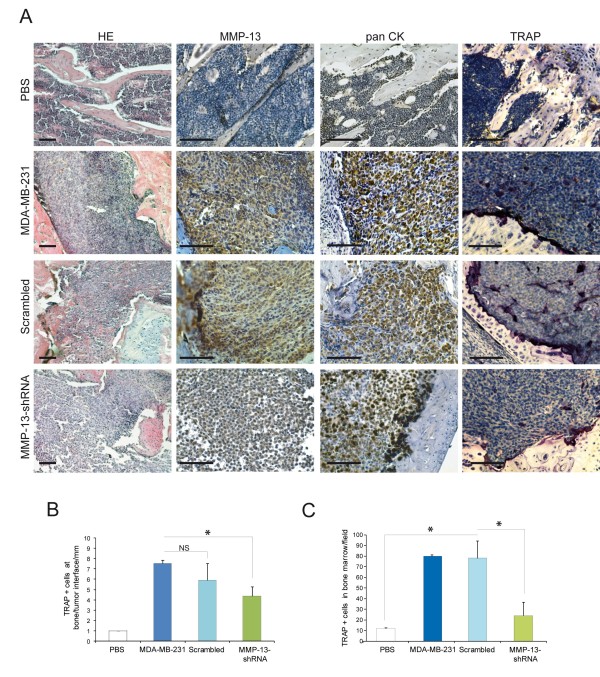
**MMP-13 expression is associated with the presence of TRAP positive cells within bone marrow and tumour**. **A**. Histology, immunohistochemical staining for MMP-13 and pan CK, and TRAP staining were performed on femurs injected with PBS, wild type, Scrambled and MMP-13 shRNA MDA-MB-231 cell clones. Representative images are shown for each conditions. The number of TRAP positive cells at tumour/bone interface (**B**) and in the bone marrow infiltrated by tumour cells (**C**) are reported. Six sections of 10 × fields of four mice for each treatment were examined. Data are expressed as mean ± SD. *P < 0.05. Scale bars = 100 μm.

WT and transfected cells were inoculated into the femurs of six-week-old nude mice and after one month ultrasound ecography and CT scans were performed to evaluate tumour mass and extent of skeletal erosion, respectively. In accord with the *in vitro *growth curves, the *in vivo *growth of MDA-MB-231 was independent of MMP-13 expression since the tumour masses developed from the different clones injected were of similar size (Figure 6A-C). On the contrary, the CT analysis confirmed the role of MMP-13 produced by tumour cells in osteolysis. In accord with the levels of MMP-13 expression in the tumours transfected with the different shRNAs (Figure 6B), the extent of bone erosion was much higher in lesions produced by WT and scrambled MMP-13 cells than that of MMP-13 shRNA clones (Figure 6D, E,Additional file 3, Figure S1). The immunohistochemical analysis and TRAP staining of mouse femurs showed that the expression of MMP-13 directly correlated with the number of TRAP positive cells (Figure 7A) that were present not only in the proximity of bone erosion (Figure 7B) but also in bone marrow (Figure 7C).

## Discussion

A key finding of the present study is that MMP-13 plays a crucial role in the microenvironment of bone metastases. MMP-13 released by breast tumour cells following stimulation with IL-8 or PTHrP played an amplifier role in the bone metastatic microenvironment by increasing and sustaining the erosion process of OCs. MMP-13 enhanced differentiation of pre-OCs, activated pro-MMP-9 released from OCs and cleaved galectin-3 expressed on OC cell surface, thus blocking its inhibitory activity on osteoclastogenesis. It has been previously reported that up-regulation of MMP-13 in mouse bone lesions is important for regulation of tumour-induced osteolysis. Knockdown of MMP-13 expression led to a significant reduction in the number of activated OCs and hence bone destruction [14]. Our present data, based on the use of specific MMP-13 shRNA in *in vitro *and *in vivo *experiments, identify a novel specific function of this enzyme in OC precursor differentiation. The relevance of the present experimental model data was supported by the finding that MMP-13 and MT1-MMP were co-expressed in human metastatic breast tumour foci in the bone.

MMP-13 is regulated by several pro-inflammatory factors, such as IL-1 and TNF-α [4]. Here we showed that MMP-13 was up-regulated in MDA-MB-231 cells also by IL-8. This chemokine attracts monocytes and OC precursors and promotes angiogenesis. In the metastatic inflammatory bone microenvironment IL-8 increases OC motility to new resorption sites [27]. Several studies have shown that under severe inflammatory conditions the elevated concentrations of MMP-13 are often matched by increased levels of interleukins, especially IL-8 [18,28-30]. This cytokine was suggested to regulate MMP-13 secretion in osteoarthritic articular chondrocytes. We found that the addition of CM obtained from MDA-MB-231 cells after IL-8 treatment to pre-OC cultures led to the increased number and size of OCs and to a more pronounced bone resorption.

Alteration in migration properties is perceived to play a pivotal role in the multi-step process that impacts on tumour cell-organ tropism and colonization. Thus, MDA-MB-231 cells very strongly adhered to both fibronectin and collagens and this could at least in part explain the tropism of breast tumour cells for bone, that is the richest tissue in collagen type I and III; however, migration on collagen type I was increased to a much greater extent by IL-8 compared to migration on fibronectin. The fact that IL-8-stimulated MDA-MB-231 cells migrated more on collagens and also were able to produce higher levels of MMP-13, sustains the hypothesis that bone microenvironment favours the production of lytic enzymes through both inflammatory cytokines and ECM components of the bone stroma.

Consistent with the findings that MMPs, produced by tumour cells, enhance OC degradation by prior removal of the overlying unmineralized layer [31], MMP-13 released by PTHrP or IL-8 primed MDA-MB-231 cells affected bone resorption (that is, OC activation and function), an activity that was fully inhibited by GM6001 and partially by CL-82198. It has been already demonstrated that MMP-9 stimulates unmineralized cartilage degradation in the presence of MMP-13 [20,23]. By the use of CL-82198, we confirmed a convergent role of MMP-9 and -13 in bone degradation. OC size is directly linked to resorptive activity [32] and the presence of MMP-13 resulted in the generation of OCs larger in size and displaying a greater resorption capacity. The MMP-13 silencing persisted also following *in vivo *inoculation, and resulted not only in a diminished bone erosion in the presence of tumour masses of similar size but also in a significant reduction of TRAP positive cells in bone marrow and within the tumour masses.

The role of MMP-13 on osteoclastogenesis could be explained as a cooperative effect with MMP-9. Among MMPs, the principal player is MMP-9 secreted by monocytes and OCs with MMP-13 derived from tumour cells acting as modulator in some specific steps of the differentiation process. MMP-13 regulates the activation of pre-MMP-9, which recruits OCs during development of long bones [23]. Mechanistically, this is an important step since the ensuing cleavage of galectin-3, a known suppressor of osteoclastogenesis [21], reduces its inhibitory function. The finding that galectin-3 is a substrate of MMP-13 *in vitro *[22] implies that MMP-13 could cleave galectin-3 expressed on OC precursors to counter its inhibitory effect *in vivo *but this mechanism remains a matter of speculation. Consistent with this hypothesis, degradation of galectin-3 became more evident *in vitro *following the addition of CM containing larger amounts of MMP-13. Another explanation for MMP-13 effect on osteoclastogenesis could be an indirect action on osteoblasts; it is often thought that MMPs and other osteogenic factors secreted by breast cancer cells activate osteoclasts through osteoblasts by changing the expression of RANKL and/or OPG [33]. Whether this possibility could explain the MMP-13 effect remains to be demonstrated *in vivo*.

## Conclusions

**Figure 8:**
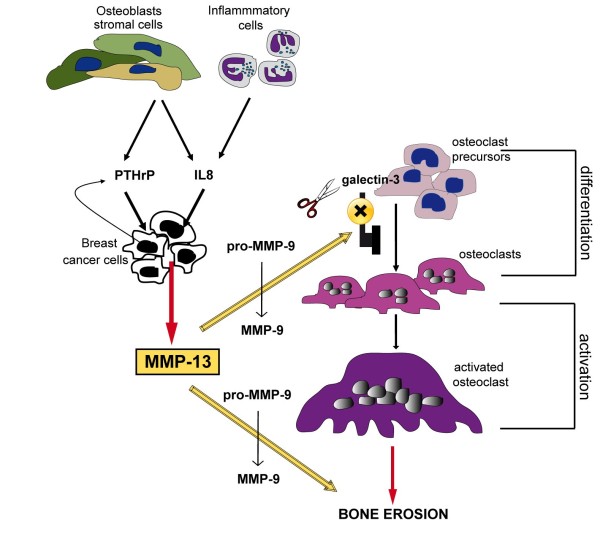
**Proposed model for the role of MMP-13 in lytic bone metastases**. MMP-13, secreted by breast tumour cells, after stimulation of ECM components or factors (IL-8, produced by inflammatory cells or osteoblasts, or PTHrP, secreted by tumour and stromal cells), could contribute to osteolysis cooperating with enzymes secreted by OCs (in particular MMP-9 present in the bone microenvironment) for matrix degradation. The effect on bone erosion could be enhanced by the indirect action of MMP-13 on the differentiation of pre-OCs and their activation. MMP-13 activates pro-MMP-9 which is known to recruit OCs and alone or together with MMP-9 cleaves galectin-3, thus blocking its inhibitory effect on osteoclastogenesis.

Several key cell types are involved in breast carcinoma bone metastasis: cancer and inflammatory cells, osteoblasts and OCs. We suggest that IL-8 and/or PTHrP produced by inflammatory cells or osteoblasts stimulate secretion of MMP-13 by breast tumour cells; MMP-13 then indirectly induces OC differentiation by activating pro-MMP-9 that, together with MMP-13 itself, could contribute to cleave the osteoclastogenesis inhibitor galectin-3, and cooperates with MMP-9 to directly degrade bone matrix (Figure 8). Clinical trials designed to test the efficacy of biologically active MMP inhibitors in a range of tumour types have been disappointing but not entirely unexpected considering all the diverse functions of the various MMPs, since only non-selective MMP inhibitor drugs entered trials. Based on the present study, a clinically usable specific MMP-13 inhibitor could be suggested as a new anti-resorptive therapeutic agent.

## Abbreviations

CM: conditioned media; DAB: diaminobenzidine; ECM: extracellular matrix; MMP: metalloproteinase; OC: osteoclast; PBMCs: peripheral blood mononuclear cells; PBS: phosphate buffered saline; TBS: tris-buffered saline; TRAP: Tartrate-Resistant Acid Phosphatase positive.

## Competing interests

The authors declare that they have no competing interests.

## Authors' contributions

EP and MS performed cell culture experiments, *in vivo *experiments and helped to write the manuscript. MP established MMP-13 silenced clones. BW performed Western blotting analyses, while IA and LB carried out computed tomography. EB produced and analysed ultrasound images. CT carried out immunohistochemical analyses on human samples. AC helped to design the experiments and PS conceived the study, designed the experiments, coordinated the studies and drafted the manuscript. All authors read and approved the final manuscript.

## Supplementary Material

Additional file 1 **Adhesion and migration profiles of MDA-MB-231 and MCF7 cells**. Additional information on cancer cell lines. Click here for file

Additional file 4 **Supplementary materials and methods**. Additional information on experimental conditions. Click here for file

Additional file 2 ***In vitro *behaviour of MMP-13 silenced cells**. Additional information on MMP-13 silenced cell properties. Click here for file

Additional file 3 **Histological appearance of bone destruction at low magnification**. Representative composed images of mouse femurs after injection of cell clones. Click here for file
