# Supplementary material for: MMP-13 stimulates osteoclast differentiation and activation in tumour breast bone metastases
Source: Breast Cancer Res. 2011 Oct 27;13(5):R105. doi: 10.1186/bcr3047 (PMC3262218; doi:10.1186/bcr3047)
Supplement: Additional file 1 — Adhesion and migration profiles of MDA-MB-231 and MCF7 cells. Additional information on cancer cell lines. [file bcr3047-S1.PDF]

ADDITIONAL FILE 1

Figure S1

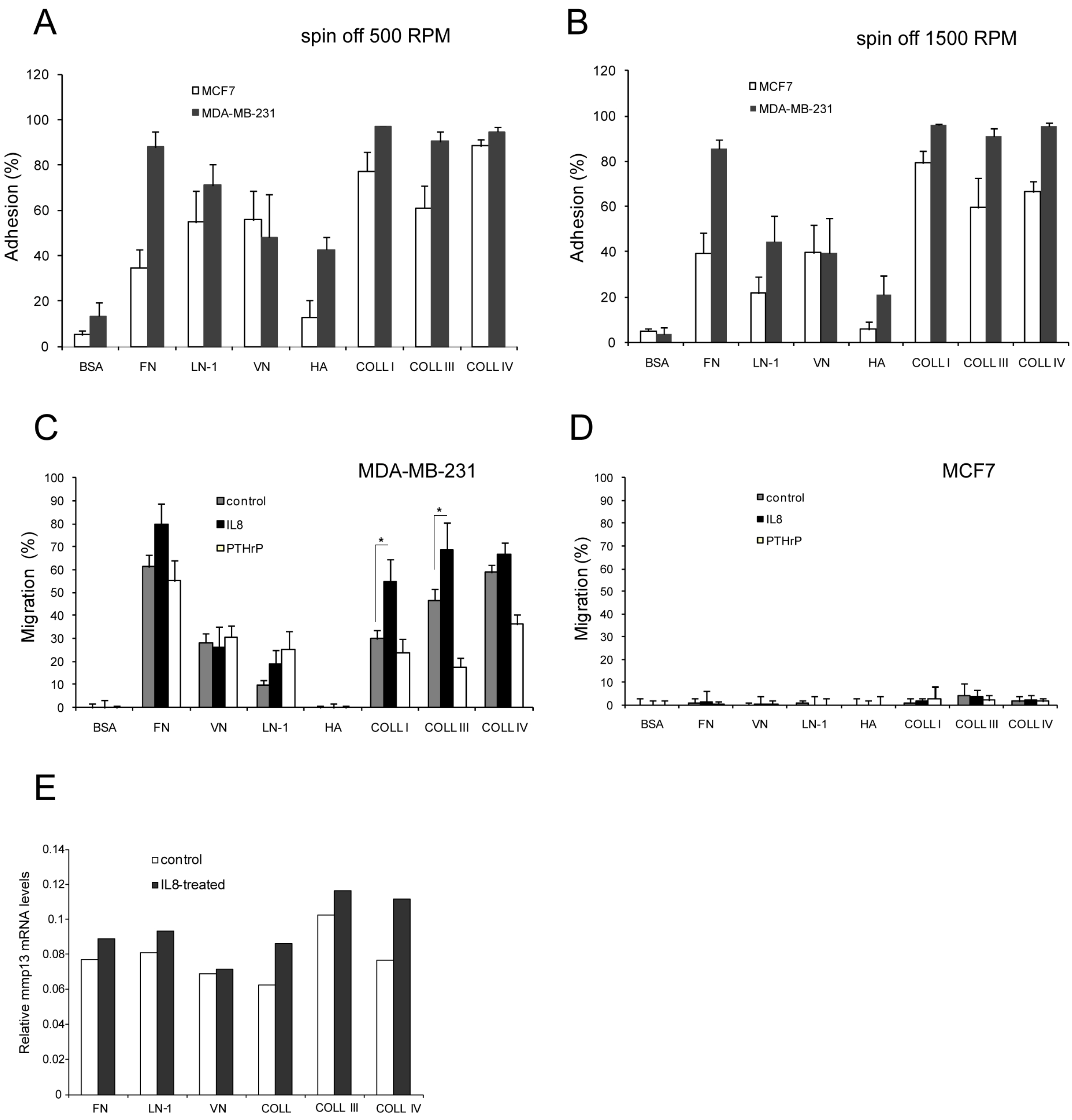

Figure S1. Adhesion and migration profiles of MDA-MB-231 and MCF7 cells. For adhesion strength two different spin off (see Additional file 4 for supplemental Materials and Methods) were applied (A and B). The time-course of haptotaxis of unstimulated or IL8/PTHrP-stimulated cells was monitored. The graphs represent cell haptotaxis expressed as the percentage of migrated cells out of total cells after 5 hours of monitoring (C and D). E, quantitative RT-PCR analysis of MMP-13 expression (related to one experiment with n = 3 replicates) after adhesion and stimulation with IL8. Cells were allowed to adhered to ECM substrates for 24 h in serum free medium and then processed for RT-PCR analysis.
