# Supplementary material for: MMP-13 stimulates osteoclast differentiation and activation in tumour breast bone metastases
Source: Breast Cancer Res. 2011 Oct 27;13(5):R105. doi: 10.1186/bcr3047 (PMC3262218; doi:10.1186/bcr3047)
Supplement: Additional file 4 — Supplementary materials and methods. Additional information on experimental conditions. [file bcr3047-S4.PDF]

ADDITIONAL FILE 4

Supplementary materials and methods

ECM molecules. Fibronectin (FN), hyaluronic acid (HA), collagen (Coll) type III and Coll type IV were purchased from Sigma; vitronectin (VN) was purified from human plasma according to the procedure of Yatohgo et al. [1]. Rat tail Coll type I was obtained from BD Biosciences. Native laminin (LN)-1-nidogen complex from EHS mouse tumour was obtained as previously described [2].

Adhesion and migration assays. CAFCA (Centrifugal Assay for Fluorescence based Cell Adhesion) and FATIMA (Fluorescence-Assisted Transmigration Invasion and Motility Assay) assays were performed as previously described for studying cell adhesion and migration behaviour, respectively [3]. For CAFCA assay six well strips of flexible polyvinyl chloride were coated with ECM molecules (20 µg/ml). Cells were labelled with the vital fluorochrome calcein AM (Molecular Probes, Invitrogen S.R.L, Milan, Italy) for 15 minutes at 37℃ and then aliquoted into the bottom miniplat es, which were centrifuged to synchronize the contact of the cells with the substrate. The miniplates were then incubated for 20 minutes at 37℃ and subsequently mounted tog ether with a similar miniplate to create communicating chambers for subsequent reverse centrifugation. The relative number of cells bound to the substrate (i.e. remaining in the wells of the bottom miniplates) and cells that fall to bind to the substrate (i.e. remaining in the wells of the top miniplates) was estimated by top/bottom fluorescence detection in a computer interfaced GeniusPlus microplate reader (Tecan, Italy). The procedure for FATIMA assay is based on the use of Transwell-like inserts carrying fluorescence-shielding porous PET membranes (polycarbonate-like material with 8 µm pores) HTS FluoroBlokTM inserts (Becton-Dickinson, Falcon). Membranes were coated (20 µg/ml in 50 µl) on the underside with the various ECM molecules. Cells were fluorescently tagged with the lipophilic dye DiI

(Molecular Probes) used at a final concentration of 5 µg/ml for 10-15 min at 37℃. The cells were then added to the upperside of the inserts (2 x 10^5 cells/insert). Migratory behaviour of the cells was then monitored at different time intervals by independent fluorescence detection from the top (corresponding to non-transmigrated cells) and bottom (corresponding to transmigrated cells) side of the membrane using the computer-interfaced GENios Plus microplate fluorometer (Tecan).

Quantitative Real-time PCR. Total RNA was extracted using Isol-RNA Lysis Reagent (5 PRIME, Gmbh, Hamburg, Germany). The RT reactions were carried out with reagents purchased from Promega, Madison, WI, USA). The RT-PCR primers were: MMP-13 left 5'-CCAGTCTCCGAGGAGAAACA-3', MMP-13 right 5'-AAAAACAGCTCCGCATCAAC-3'; endogenous control gene polymerase II left 5'-GCAAATTCACCAAGAGAGACG-3' right 5'-CACGTCGACAGGAACATCAG-3'. Quantitative Real Time PCR was performed using the AB 7700 Real Time PCR System (Applied Biosystems, Carlsbad, CA, USA).

Proliferation assay. To quantitatively monitor cell proliferation in real-time we adopted the innovative technology provided by the RTCA DP instrument (Roche Diagnostics GmbH, Germany). The strategy is based on continuous, quantitative monitoring of cells as they adhere and proliferate by measuring electrical impedance [4]. The change in impedance caused by cell attachment and proliferation is expressed as Cell Index (CI), that is an arbitrary measurement defined as (Rn − Rb)/15, where Rb is the background impedance of the well measured with medium alone and Rn is the impedance of the well measured at any time (t) with cells present. The CI is thus a reflection of overall cell number, attachment quality and cell morphology which can change as a function of time. MDA-MB-231 cells were starved overnight and seeded in E-plates 96 at 20.000 cells/well. The E-plates 96 were monitored at different time-interval for 80 hours. Data analysis was carried out using RTCA Software 1.2 supplied with the instrument.

1. Yatohgo T, Izumi M, Kashiwagi H, Hayashi M: **Novel purification of vitronectin from human plasma by heparin affinity chromatography**. *Cell Struct Funct* 1988, **13**:281-92.

2. Spessotto P, Yin Z, Magro G, Deutzmann R, Chiu A, Colombatti A, Perris R. **Laminin isoforms 8 and 10 are primary components of the subendothelial basement membrane promoting interaction with neoplastic lymphocytes**. *Cancer Res* 2001, 61:339-47.

3. Spessotto P, Lacrima K, Nicolosi PA, Pivetta E, Scapolan M, Perris R: **Fluorescence-based assays for in vitro analysis of cell adhesion and migration**. *Methods Mol Biol* 2009, **522**: 221-50.

4. Xing JZ, Zhu L, Jackson JA, Gabos S, Sun XJ, Wang XB, Xu X: **Dynamic monitoring of cytotoxicity on microelectronic sensors**. *Chem Res Toxicol* 2005, **18:** 154-61.
