# Supplementary material for: MMP-13 stimulates osteoclast differentiation and activation in tumour breast bone metastases
Source: Breast Cancer Res. 2011 Oct 27;13(5):R105. doi: 10.1186/bcr3047 (PMC3262218; doi:10.1186/bcr3047)
Supplement: Additional file 2 — In vitro behaviour of MMP-13 silenced cells. Additional information on MMP-13 silenced cell properties. [file bcr3047-S2.PDF]

ADDITIONAL FILE 2

Figure S1

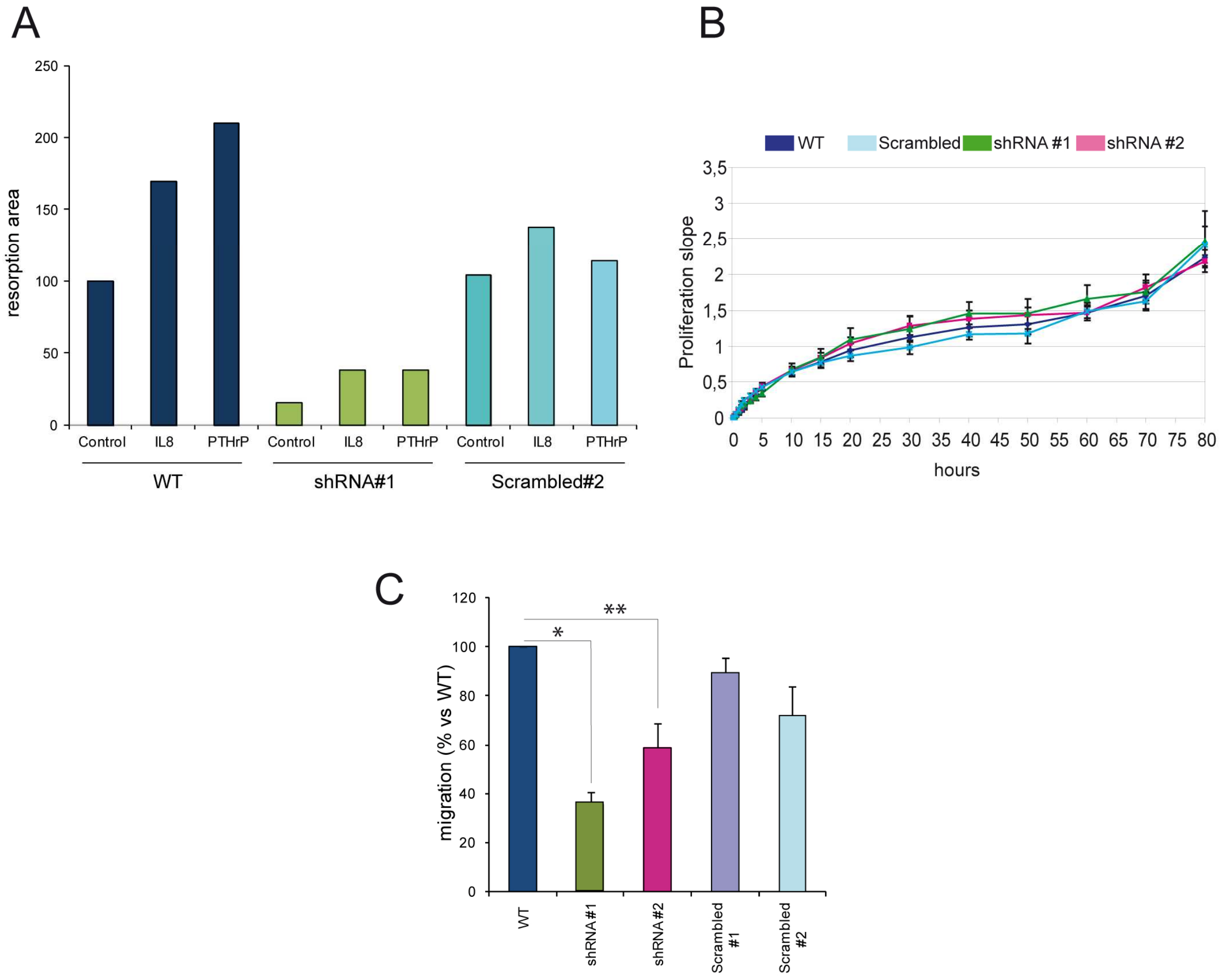

Figure S1. In vitro behaviour of MMP-13 silenced cells. A, PBMCs were seeded on Biocoat matrices to evaluate resorption activity. Cells were primed with M-CSF and RANKL for three days and then further cultured for four days with the addition of CM from WT, shRNA or Scrambled MDA-MB-231 cells pre-stimulated or not with IL8 or PTHrP. The quantification of resorption area was expressed as the percentage vs control cells (WT).

The graph reports the mean values obtained from two experiments. B, Dynamic monitoring of proliferation rates expressed as cell index using the XCELLigence system of wild type (WT), Scrambled and two clones (shRNA #1 and shRNA #2) of MMP-13 silenced cells (see Additional file 4 for supplemental Materials and Methods). Cell index was calculated as the mean ± SD from n = 3 experiments with n = 6 replicates. C, haptotaxis of scrambled and shRNA cell clones migrating for five hours toward collagen type I expressed as percentage vs control cells (WT).
