# Supplementary material for: MMP-13 stimulates osteoclast differentiation and activation in tumour breast bone metastases
Source: Breast Cancer Res. 2011 Oct 27;13(5):R105. doi: 10.1186/bcr3047 (PMC3262218; doi:10.1186/bcr3047)
Supplement: Additional file 3 — Histological appearance of bone destruction at low magnification. Representative composed images of mouse femurs after injection of cell clones. [file bcr3047-S3.PDF]

ADDITIONAL FILE 3

Figure S1

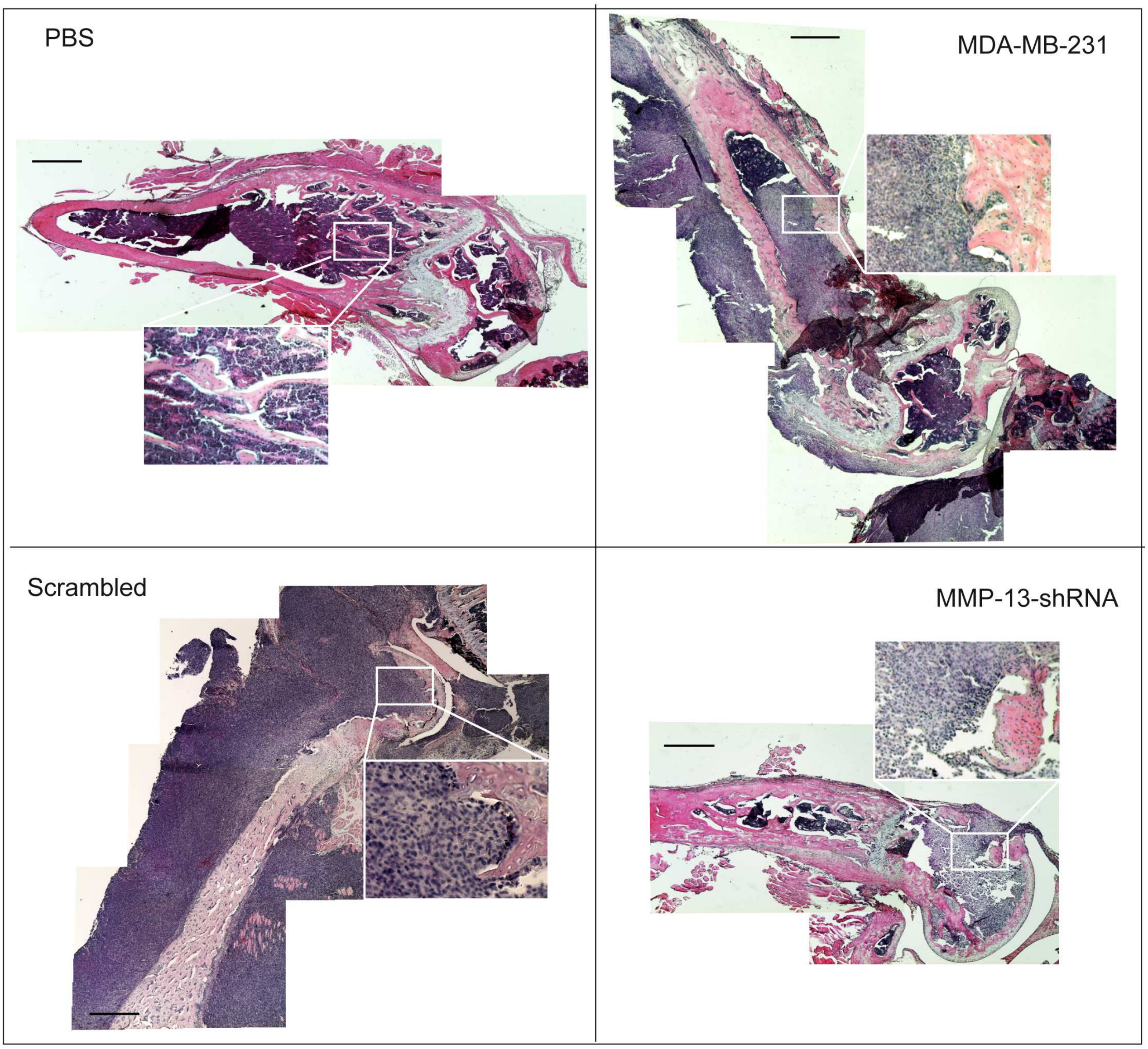

Figure S1. Histological appearance of bone destruction at low magnification. Representative composed images of femurs injected with PBS, wild type (MDA-MB-231), scrambled and shRNA MDA-MB-231 cell clones were obtained after the automatic alignment provided by Adobe Photoshop CS4 software of acquired fields with a 5X objective. The insets are higher magnification of fields reported in Figure 7A (except for Scrambled sample). Scale bar = 500 µm.
